# Palliative distal gastrectomy offers no survival benefit over gastrojejunostomy for gastric cancer with outlet obstruction: retrospective analysis of an 11-year experience

**DOI:** 10.1186/1477-7819-12-364

**Published:** 2014-11-29

**Authors:** Yasuhiro Okumura, Hiroharu Yamashita, Susumu Aikou, Koichi Yagi, Yukinori Yamagata, Masato Nishida, Kazuhiko Mori, Sachiyo Nomura, Joji Kitayama, Toshiaki Watanabe, Yasuyuki Seto

**Affiliations:** Department of Gastrointestinal Surgery, The University of Tokyo, 7-3-1 Hongo, Bunkyo-ku, Tokyo, 113-8655 Japan; Surgical Oncology, Graduate School of Medicine, The University of Tokyo, 7-3-1 Hongo, Bunkyo-ku, Tokyo, 113-8655 Japan

**Keywords:** Gastric cancer with outlet obstruction, Gastrojejunostomy, Palliative distal gastrectomy

## Abstract

**Background:**

Either palliative distal gastrectomy or gastrojejunostomy are the initial treatment options for locally advanced gastric cancer with outlet obstruction when curative-intent resection is not feasible. Since chemotherapy is the mainstay for unresectable gastric cancer, the clinical value of palliative distal gastrectomy is controversial.

**Methods:**

We retrospectively reviewed the clinical data of patients with gastric cancer with outlet obstruction treated at our institution between January 2002 and December 2012. We compared the clinical outcomes of palliative distal gastrectomy with those of gastrojejunostomy patients and the factors affecting overall survival were evaluated.

**Results:**

Elective palliative distal gastrectomy and gastrojejunostomy were performed in 18 and 25 patients, respectively. The median overall survival times in the gastrojejunostomy and palliative distal gastrectomy groups were statistically equivalent at 8.8 and 8.3 months, respectively (*P* = 0.73), despite the more locally advanced tumors in the gastrojejunostomy as compared with the palliative distal gastrectomy group. A multivariate Cox regression analysis showed absence of postoperative chemotherapy and higher postoperative complication grade to be associated with worse clinical outcomes.

**Conclusions:**

Palliative distal gastrectomy offers neither survival nor palliative benefit as compared to gastrojejunostomy. Minimizing the morbidity of intervention for outlet obstruction, followed by chemotherapy, appears to be the optimal initial strategy for incurable gastric cancer with outlet obstruction.

## Background

Gastric cancer with outlet obstruction (GCOO) is a locally advanced malignancy characterized by tumor ingrowth. GCOO is also associated with outward tumor growth and invasion, as evidenced by 47% of GCOO showing direct invasion of adjacent organs [1]. More importantly, GCOO frequently metastasizes to lymph nodes (93%), the peritoneum (34%), and the liver (15%) [1], suggesting a systemically advanced tumor. Patients with GCOO had worse clinical outcomes than those without outlet obstruction even after curative resection [2].

Although chemotherapy is generally the mainstay for treatment of advanced disease, the symptoms associated with GCOO, such as nausea, vomiting, and poor nutritional status due to impaired food intake, hamper administration of oral regimens. In fact, most clinical trials of palliative chemotherapy for advanced/recurrent gastric cancer exclude these patients [3–5] as chemotherapeutic regimens commonly consist of the oral fluoropyrimidine derivative S-1/capecitabine, alone or in combination with other drugs. Therefore, the initial management step for outlet obstruction should be urgent surgical or endoscopic relief of obstructive symptoms, followed by systemic chemotherapy.

When we select a surgical approach for a GCOO patient and then identify a factor rendering the tumor incurable, based on inspection by laparotomy/laparoscopy, what is the optimal approach? Resection reportedly confers a 3 to 5 month survival benefit over non-resection and 8 months overall even in the palliative setting, such that palliative gastrectomy, if feasible, was long considered to be the best option for incurable GCOO [1, 6, 7]. However, this view has changed in the last decade. Recently, median survival times (MST) after palliative gastrectomy have gradually been increasing and have now reached nearly 1 year according to the literature [8–11]. This survival improvement is likely attributable to the recent development of chemotherapy for unresectable advanced/recurrent gastric cancer providing MST of 1 year or longer [5, 12, 13]. It is noteworthy that the presence of postoperative chemotherapy is a powerful indicator of prolonged survival after cytoreductive palliative gastrectomy [8–11].

However, according to the study results currently available, debate continues as to whether non-curative gastrectomy followed by systemic chemotherapy or chemotherapy without gastrectomy is most appropriate for incurable GCOO. We evaluated clinical outcomes using our cohort with incurable GCOO to determine the optimal procedure for this disease entity in the present study.

## Methods

### Patient population

Between January 2002 and December 2012, a total of 1,531 patients with gastric cancer were treated at the Department of Surgery, The University of Tokyo. Among these 1,531 patients, 97 (6.3%) were diagnosed as having GCOO, and we retrospectively reviewed their clinical records. GCOO is defined as an advanced gastric cancer arising from the distal third of the stomach with symptoms including nausea, vomiting, or inability to consume a regular diet. Even if patients did not suffer these symptoms, endoscopically-proven massive food residue in the stomach or inability to pass the endoscope into the duodenum is considered to represent GCOO. Five patients with outlet obstruction due to progressive primary disease after chemotherapy failure were excluded. The remaining 92 patients constituted the entire cohort with GCOO.

**Figure 1:**
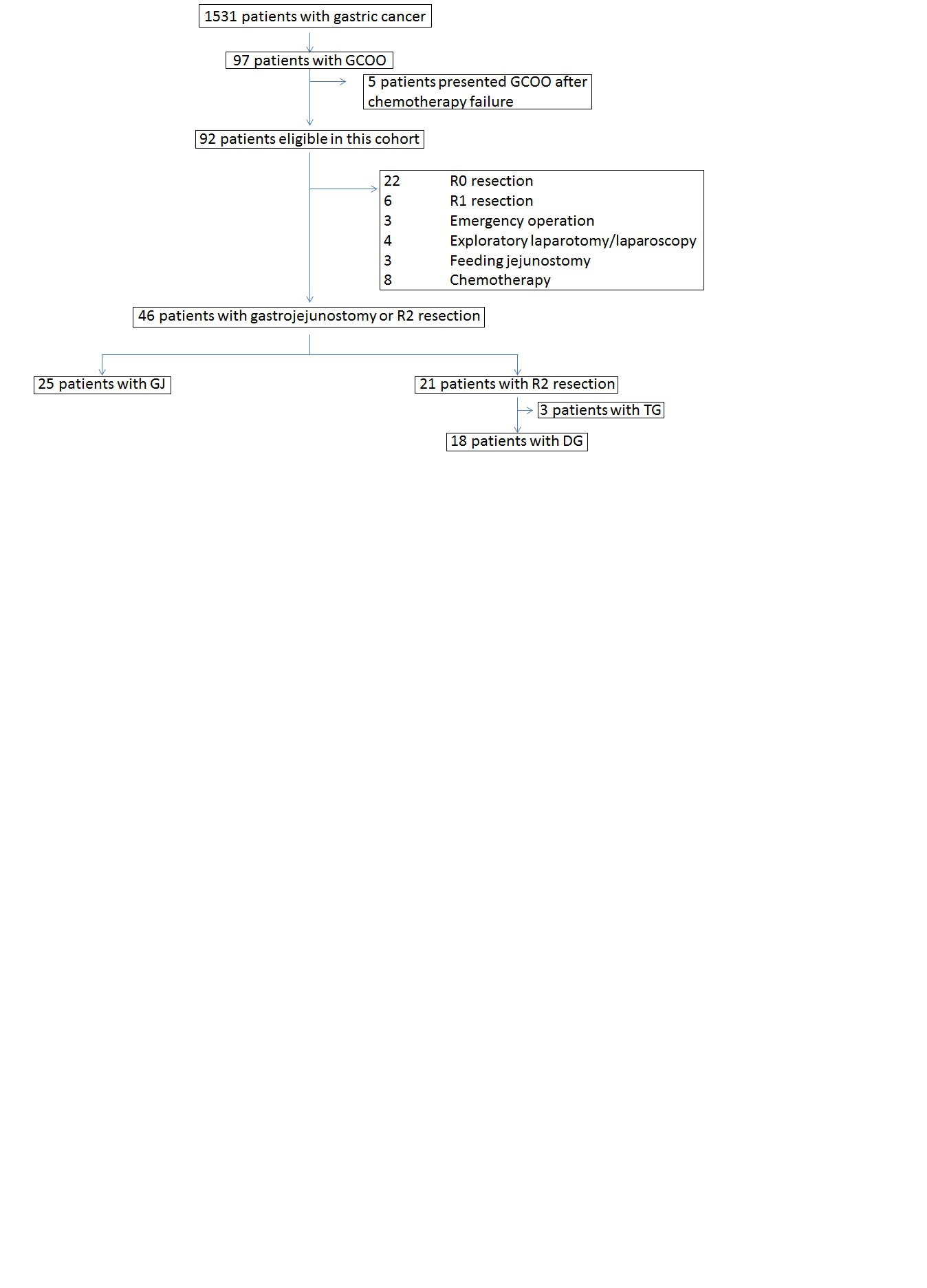
**CONSORT diagram for this study.** We retrospectively reviewed 25 gastrojejunostomy cases and 18 palliative distal gastrectomy cases. DG, Distal gastrectomy; GCOO, Gastric cancer with outlet obstruction; GJ, Gastrojejunostomy; TG, Total gastrectomy.

In our cohort, R0 and R1 resections were achieved in 22 and 6 patients, respectively. Among the remaining 64, 3 patients underwent emergency operations (1 gastrectomy and 2 gastrojejunostomy (GJ)) for perforation or uncontrolled bleeding from a gastric cancer. Exploratory laparotomy/staging laparoscopy and feeding jejunostomy were performed for 4 and 3 patients, respectively. Chemotherapy was the initial treatment in 8 patients. Total gastrectomy was performed in 3 patients with palliative intent. The remaining 43 patients receiving GJ or palliative distal gastrectomy (PDG) were evaluated in this study (Figure 1). Our protocol was approved by the ethics committee of the faculty of medicine, the University of Tokyo.

GJ and PDG were performed in 25 and 18 patients, respectively. Postoperative complications were classified according to the Clavien-Dindo system [14]. Complications of grade 2 or higher were defined as postoperative morbidity [15]. We also assessed oral intake employing the gastric outlet obstruction scoring system (GOOSS), where 0 = no oral intake, 1 = liquids only, 2 = soft solids, and 3 = low-residue or full diet [16]. ΔGOOSS was calculated by subtracting preoperative GOOSS from postoperative GOOSS. Performance status (PS) was assessed employing the European Clinical Oncology Group (ECOG) scale. Postoperative chemotherapy was performed after sufficient postoperative recovery. S-1 combined with cisplatin (CDDP) is the first choice for patients with a favorable postoperative course and adequate renal function. Otherwise, basically S-1, alone or combined with a taxane derivative, was selected.

We conducted tumor staging according to the Union for International Cancer Control TNM staging system for the stomach [17]. We evaluated tumor histology according to the Lauren classification [18]. Well and moderately differentiated tubular adenocarcinoma, papillary adenocarcinoma, and solid type poorly differentiated adenocarcinoma were classified as intestinal-type carcinomas; non-solid type poorly differentiated adenocarcinoma, signet ring cell carcinoma, and mucinous carcinoma were classified as diffuse-type carcinomas. Other tumors were classified separately.

### Statistical analysis

Statistical analyses were carried out using JMP 10.0.2 (SAS institute, Cary, NC, USA). The overall survival rates were calculated from the operation date. Kaplan-Meier survival curves were used to estimate the impact of each procedure in our series, and the log-rank test was employed for comparisons.

Differences in patient characteristics (sex, histology, direct invasion of adjacent organs (T4b), liver metastasis (H), peritoneal metastasis (P), stage, and presence/absence of postoperative morbidity, and chemotherapy) as categorical variables were compared between the GJ and PDG groups by the *χ*^2^ test. The Wilcoxon test was conducted for continuous variables including age, ECOG PS, preoperative GOOSS, operative time, intraoperative blood loss, and ΔGOOSS. A Cox proportional-hazards analysis was performed to identify independent prognostic factors among the variables (sex, age, operative procedure (GJ or PDG), ECOG PS, histology (intestinal or diffuse type adenocarcinoma), preoperative GOOSS, presence/absence of postoperative chemotherapy, postoperative complications, T4b, H, and P) for overall survival. *P* values less than 0.05 were considered significant.

## Results

Sixty of the 92 patients (65%) with GCOO had Stage IV disease, and the incidences of T4b, H, and P were 40%, 15%, and 40%, respectively. Gastrectomy was performed in 49 cases including 22 R0, 6 R1, and 21 R2 resections.

The clinical characteristics of the 43 patients undergoing elective surgery with GJ or PDG are summarized in Table 1. Sex, age, stage, and ECOG PS showed no statistically significant differences between the GJ and PDG groups. Preoperative GOOSS was significantly worse (*P* = 0.02) and the majority required total parenteral nutrition (64% vs. 28%, *P* = 0.02) in the GJ group. Intestinal type was the dominant histology in the GJ group but not in the PDG group (64% vs. 33%, *P* = 0.05). Although the rates of H, P, and distant lymph node metastasis were equivalent in these two groups, 88% of patients with GJ had T4b tumors while only 11% of the PDG group had T4b tumors (*P* <0.0001).

**Table 1:** **Characteristics of 43 patients undergoing gastrojejunostomy or palliative distal gastrectomy**

	Gastrojejunostomy	Gastrectomy	***P*** value
	(n = 25)	(n = 18)	
Sex			
Male:female	17:8	10:8	0.40
Age			
Median (range)	70 (52–82)	74 (44–88)	0.42
Histology			
Intestinal:diffuse	16:9	6:12	0.05
Staging			
Direct invasion to neighboring organ(s)	22	2	<0.0001
Peritoneal metastasis	19	13	0.78
Liver metastasis	4	3	0.95
Distant lymph node metastasis	6	4	0.89
Stage			
IIIC:IV	4:21	1:17	0.29
ECOG PS			
0:1:2:3	13:10:2:0	11:3:1:3	0.87
Preoperative GOOSS			
0:1:2:3	15:8:1:1	6:5:1:6	0.02
Number of patients with preoperative TPN	16	5	0.02

ECOG, European Clinical Oncology Group; GOOSS, Gastric outlet obstruction scoring system; TPN, Total parenteral nutrition.

All GJ procedures were of the stomach-partitioning type. All procedures were performed by an open approach. Operative times did not differ significantly (165 vs. 189 min, *P* = 0.08), while intraoperative blood loss was significantly lower (86 mL vs. 215 mL, *P* = 0.02) in the GJ group (Table 2). ΔGOOSS and number of patients given postoperative chemotherapy did not differ significantly. Eight patients (32%) in the GJ group had complications, including two with anastomotic stricture, two with anorexia, three with catheter infection, and one with another complication. Meanwhile, two patients (11%) in the PDG group had complications, including anorexia and urinary tract infection. Morbidity rates did not differ significantly between the two groups (*P* = 0.10).

**Table 2:** **Early outcomes and chemotherapy after gastrojejunostomy and palliative gastrectomy**

	Gastrojejunostomy	Gastrectomy	***P*** value
	(n = 25)	(n = 18)	
Operative time (min)	165 (102–365)	189 (94–364)	0.08
Intraoperative blood loss (mL)	86 (0–950)	215 (42–790)	0.02
ΔGOOSS			
Median (range)	2 (0–3)	1 (-2–3)	0.13
Morbidity	8	2	0.10
Anastomotic stricture	2	0	
Anastomotic leakage	0	0	
Anorexia	2	1	
Catheter infection	3	0	
Others	1	1	
Number of patients given postoperative chemotherapy	19	15	0.56
Regimens			
S-1	6	5	
S-1 + CDDP	13	6	
S-1 + Taxane(DTX/PTX)	0	2	
Other	0	2	

CDDP, Cisplatin; DTX, Docetaxel; GOOSS, Gastric outlet obstruction scoring system; PTX, Paclitaxel.

**Figure 2:**
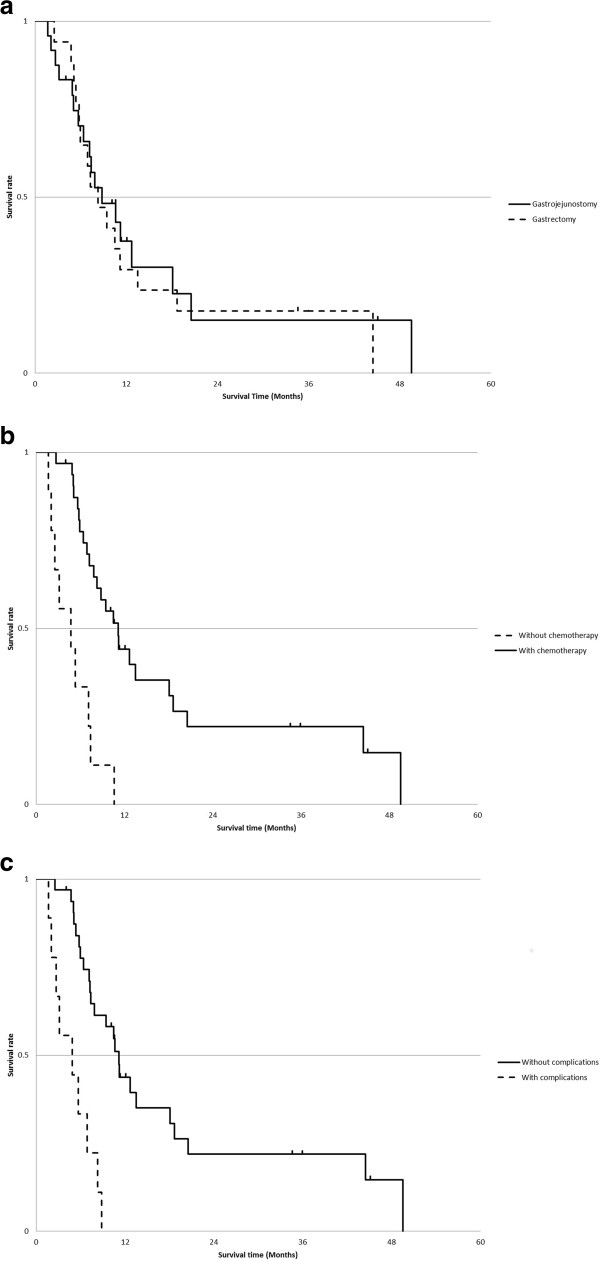
**Survival curve of 43 patients with incurable gastric cancer with outlet obstruction.** **(** **a** **)** Overall survivals after gastrojejunostomy and palliative distal gastrectomy. Overall survival did not differ between the gastrojejunostomy and palliative distal gastrectomy groups (*P* = 0.73). **(** **b** **)** Overall survivals of patients with and without postoperative chemotherapy. Patients with postoperative chemotherapy had significantly better survival than those without (*P* <0.0001). **(** **c** **)** Overall survivals of patients with and without postoperative complications. Patients who had postoperative complications had significantly worse survival than those who did not (*P* <0.0001).

Chemotherapeutic regimens are summarized in Table 2. Postoperative chemotherapy was given to 19 (76%) of the GJ and 15 (83%) of the PDG group patients. MST were 8.8 and 8.3 months in the GJ and PDG groups, respectively, and were not significantly different (*P* = 0.73, Figure 2a). Univariate analysis showed survival to be significantly related to ECOG PS, presence of postoperative chemotherapy, and absence of postoperative complications (Table 3). A multivariate Cox regression analysis showed absence of postoperative chemotherapy and presence of postoperative complications to be independently associated with poor clinical outcomes. Patients receiving postoperative chemotherapy had significantly better survival than those who did not (MST 11.2 vs. 4.7 months, *P* <0.0001, Figure 2b). MST of patients with and without postoperative complications were 4.9 and 11.2 months, respectively (*P* <0.0001, Figure 2c).

**Table 3:** **Factors predicting overall survival**

Variables	Univariate			Multivariate		
	HR	CI (95%)	***P*** value	HR	CI (95%)	***P*** value
Sex	1.73	0.79–3.59	0.16			
Age	1.41	0.69–2.96	0.35			
Operative procedure	1.13	0.56–2.28	0.73			
ECOG PS	3.01	1.08–7.22	0.04	1.78	0.54–5.40	0.33
Histology	1.12	0.55–2.30	0.76			
Preoperative GOOSS	1.22	0.51–2.62	0.63			
Postoperative chemotherapy	0.20	0.09–0.48	0.0007	0.16	0.06–0.49	0.0016
Postoperative complication(s)	3.68	1.50–8.62	0.0054	5.88	2.24–15.35	0.0005
Liver metastasis	2.43	0.95–5.49	0.06			
Peritoneal metastasis	1.04	0.51–2.13	0.92			
Direct invasion into neighboring organ(s)	1.22	0.60–2.46	0.58			

CI, Confidence interval; ECOG, European Clinical Oncology Group; GOOSS, Gastric outlet obstruction scoring system; HR, Hazard ratio.

## Discussion

In our study, GCOO showed high incidences of T4b, H, and P. Our results are mostly consistent with those of a previous report [1]. Therefore, GCOO is characterized by a high probability of Stage IV disease and has a poor prognosis. Chemotherapy is clearly the mainstay in treating this population.

In our series, surgical procedures were selected by surgeons according to the individual needs of their patients. During this period, we preferentially selected gastrectomy, if feasible, even when factors making a tumor incurable were present. This was based mainly on obtaining a possible survival benefit with R2 gastrectomy over GJ, as demonstrated in previous studies [6, 8]. In fact, there were only three tumors (14%) in the GJ group without adjacent organ infiltration, while 89% of tumors in the PDG group had this feature.

R2 gastrectomy is known to be associated with high morbidity and mortality rates as compared with R1 resection or GJ [19]. Although GJ is assumed to be a less invasive and lower morbidity alternative for GCOO, our results do not support this assumption. In our study, GJ had a shorter operative time and significantly less intraoperative blood loss, though postoperative morbidity was not better, instead actually being worse, than with PDG. There were three procedure conversions from PDG to GJ after extensive peritumoral area manipulation. This challenging situation resulted in longer operative times, larger amounts of blood loss, and more postoperative complications. Intraoperative conversions and other challenges should be avoided whenever possible and PDG cannot be recommended, at least for T4b disease, according to our present results.

Gastrectomy was long considered to basically be the best option, even for stage IV patients, in some institutes [6, 7]. Since recent advances in chemotherapy have produced high response rates and prolonged the survival of patients with gastric cancer [5, 12, 13], chemotherapy rather than cytoreductive gastrectomy has come to play an essential role in the treatment of incurable gastric cancer [8–11]. Epirubicin, oxaliplatin, and capecitabine combination therapy achieved a higher response rate (47.9%) and longer overall survival (MST, 11.2 months) than other triplet combination therapies with cisplatin or fluorouracil in the REAL-2 trial [20]. In Japan, doublet administration with S-1 plus cisplatin became the standard first-line regimen after the SPIRITS trial, demonstrating a response rate of 54% and a MST of 13 months [12]. These regimens contain the oral fluoropyrimidine derivative S-1/capecitabine, with the intake being uniquely impeded by GCOO, such that the most promising chemotherapies are not an option for patients whose initial status is GCOO. Therefore, initiation of chemotherapy, rather than an operative procedure, was associated with better survival in patients with Stage IV GCOO, in this retrospective study.

According to the multivariate Cox regression analysis, operative procedure was not a prognostic factor in patients with GCOO. Meanwhile, absence of postoperative chemotherapy and presence of postoperative complications were associated with worse clinical outcomes. Postoperative complications delayed recovery and disrupted subsequent chemotherapy, in general. In fact, 3 of the 11 patients (27%) with postoperative morbidity received no chemotherapy. Therefore, intervention without complications is essential in the treatment of unresectable GCOO.

Theoretically, endoscopic stenting is the most minimally invasive approach for outlet obstruction currently available. Self-expanding stents reportedly achieve earlier improvement of oral intake and a lower morbidity rate than surgical interventions [21–24]. Recent reports have recommended endoscopic stenting for patients with life expectancies shorter than 2 months, while GJ is preferable for those with good PS and/or longer estimated survival according to their patency duration [23–25]. Endoscopic stenting has been shown, by two randomized controlled trials comparing it with GJ, to offer better short term outcomes [26, 27]. Although the populations consisted mainly of patients with pancreatic cancer in these prior studies, endoscopic stenting might be a minimally invasive option for patients with GCOO as well.

Ohashi et al. reported the efficacy of GJ as the initial therapy for incurable GCOO [28]. The MST of patients with incurable GCOO after GJ followed by S-1-based chemotherapy was reported to be 354 days [27]. This strategy seems to be feasible and is supported by case reports describing successful treatments [29–32]. In these cases, gastrectomy with curative intent was achieved after marked tumor shrinkage in response to chemotherapy. Since endoscopic stenting is associated with a lower complication rate and earlier initiation of chemotherapy, it might be worthwhile to evaluate whether or not the bridge to surgery concept, a promising treatment strategy for obstructive colorectal cancer, is also feasible for GCOO.

The major limitations of this study are its small population size, retrospective nature, and it having been conducted in a single institution. Furthermore, no definite protocol to select either procedure yielded clear differences in patient characteristics between the two groups, especially regarding depth of the primary tumor, as was demonstrated by one retrospective study advocating palliative gastrectomy over GJ and endoscopic stenting for GCOO [33]. A prospective study excluding T4b tumors is warranted before any definite conclusions can be drawn.

## Conclusions

In conclusion, PDG offers neither survival nor palliative benefit as compared to GJ. Therefore, surgical removal should not necessarily be recommended for patients with this pathology. Intervention without complications and induction chemotherapy constitute an ideal initial approach for patients with unresectable GCOO. Chemotherapy, rather than cytoreductive gastrectomy, plays an essential role in improving the outcomes of this patient population.

## Abbreviations

CDDP: Cisplatin
ECOG: European Clinical Oncology Group
GCOO: Gastric cancer with outlet obstruction
GJ: Gastrojejunostomy
GOOSS: Gastric outlet obstruction scoring system
H: Liver metastasis
MST: Median survival times
P: Peritoneal metastasis
PDG: Palliative distal gastrectomy
PS: Performance status
T4b: Direct invasion of adjacent organs.
